# Telepharmacy in Nigeria: a narrative review

**DOI:** 10.3389/fpubh.2026.1843197

**Published:** 2026-06-17

**Authors:** Oyebode Oluwaseyi Dosunmu

**Affiliations:** Department of Clinical Pharmacy and Pharmacy Practice, Faculty of Pharmaceutical Sciences, University of Ibadan, Ibadan, Nigeria

**Keywords:** analysis, improvement, Nigeria, readiness, telepharmacy

## Abstract

Telepharmacy is being employed to deliver effective pharmaceutical services remotely, especially to rural and hard-to-reach areas. Countries have been making use of this approach effectively. The review explored the use of telepharmacy in Nigeria, the country’s readiness, and strategies for the enhancement of telepharmacy in the country. Studies conducted on telepharmacy use in Nigeria were searched on six databases, including CINAHL Plus, Embase, PsycINFO, Scopus, Web of Science, and Google Scholar. The keywords used for the search are “Nigeria” and “Telepharmacy.” The screening was done using Covidence. Ten studies were included at the end of the screening. The findings from the study show that telepharmacy use in Nigeria is in its infancy and emerging. Only Lagos State, among the States included in the review, shows comparatively greater preparedness for large-scale telepharmacy implementation, though evidence remains limited. Telepharmacy services were reported to be useful. However, various challenges have been a deterrent. The most notable of these challenges is unreliable internet connectivity and unstable power supply. It is suggested that government, professional organizations, and institutions address these challenges through systemic strategies such as improving internet infrastructure and ensuring a stable power supply. These actions can make Nigeria ready for telepharmacy and deliver effective pharmaceutical services to all locations.

## 1 Introduction

Telepharmacy is the use of telecommunication or technology, by the pharmacist, to provide pharmaceutical services remotely to patients (1–3). It is mostly employed for reaching patients or clients in areas where pharmacy services may be hard to reach or where there is insufficient pharmacy expertise, such as rural areas (2, 3). Sometimes, it can be used to make rendering pharmaceutical services convenient for both the pharmacist and patients (1). Such pharmaceutical services may include pharmaceutical counseling, drug therapy monitoring, and addressing complaints or side effects of medications being used, among others (4).

The World Health Organization (WHO), in its efforts to improve care in the healthcare system, came up with the six building blocks of health (5–7). These include health service delivery, health workforce, health information systems, access to essential medicines, health systems financing, and leadership and governance. The building blocks serve as the framework and foundation on which nations built their healthcare system for sustainability and resiliency. They ensure each of the blocks is targeted and catered for in order to elicit the desired healthcare quality outcome.

Nigeria, a developing country with thirty-six states and a Federal Capital Territory (8), uses the WHO building block as a framework for strengthening its healthcare system (9, 10). The building blocks are adapted into its National Strategic Health Development Plan towards efforts for the achievement of mandate of quality care for all, in alignment with the United Nations Sustainable Development Goal (UN SDG) 3 (11). Telepharmacy can be used as a tool for contributing to this goal of quality care for all in Nigeria. Therefore this article seeks to analyze the current situation of telepharmacy in Nigeria, discuss the readiness for full implementation of telepharmacy, and offer suggestions to the government and professional organizations on strategies to enhance telepharmacy in Nigeria.

## 2 Methods

A narrative review of the literature was conducted to explore studies on telepharmacy use in Nigeria. The keywords used are “Nigeria” and “telepharmacy.” These two primary keywords were used because they are core concepts of the highly specific topic under review. Using them only ensured search specificity for relevant and influential literature, while the inclusion of unrelated studies was minimized. Qualitative and quantitative studies were included. Previous narrative reviews were also included to understand the reported trend of telepharmacy use in Nigeria over the years and for complementing the findings from research articles in the included studies. Exclusion criteria included pre-print and studies on telemedicine that does not include pharmacists’ or patients’ participation.

Six (6) databases, including CINAHL Plus, Embase, PsycINFO, Scopus, Web of Science, and Google Scholar, were searched but CINAHL Plus elicited no result (Appendix 1). Citation searching was also conducted. There was no date restriction. Initial searches did not impose language restriction; however, only English Language studies were ultimately included. Covidence was used for the management of the review process. At the end of the screening processes, ten studies were considered eligible for review. Experts were consulted and surmised that the retrieved articles will elicit an overview information of telepharmacy use in Nigeria. Thematic analysis of the findings was conducted.

## 3 Results

The results of the characteristics of studies and findings from included studies are shown in Tables 1, 2, respectively.

**Table 1:** Characteristics of included studies.

S/N	Author, year	Setting	Method	Population
1	Chika et al., 2024 (12)		Narrative review	
2	Adesokun et al., 2024 (19)	Osun State	Prospective cohort study	Type 2 diabetes patients
3	Nduka et al., 2023 (18)	Anambra State	Cross-sectional survey	Community pharmacists and patients
4	Ogugua et al., 2023 (14)	FCT	Cross-sectional survey	Community pharmacists’ clients
5	Oliorah et al., 2022 (15)	Lagos State	Cross-sectional survey	Community pharmacists
6	Anosike et al., 2020 (13)	Enugu State	Cross-sectional survey	Community pharmacies’ patients
7	Cremers et al., 2019 (16)	Lagos State	Qualitative study	Cardiologists, Community pharmacists, Hypertensive patients
8	Ola-Olorun et al., 2019 (17)	Osun State	Cross-sectional survey	Community pharmacists
9	Nelissen et al., 2018 (4)	Lagos State	Cross-sectional survey	Community pharmacists
10	Igharo, 2013 (20)		Narrative review	

**Table 2:** Challenges faced by telepharmacy.

WHO building block	Challenges
Health service delivery	Physicians’ concern for pharmacists taking over of their responsibility (16)
Health workforce	Healthcare workers shortage and brain drain (4, 12)
Health information system	Available digital app not user friendly (4) Unreliable internet connectivity (4, 12, 15, 16) Unstable power supply (12) Data privacy concern (12) High installation cost (15) Lack of ICT resources (15)
Access to medicines	High cost of medicine making it difficult to afford telepharmacy services (20)
Health system financing	Financial constraints (4, 12, 13, 16, 19)
Leadership and governance	No adaptable regulatory framework/absence of telepharmacy policy (12)

Most of the studies (6) were reported during or after the COVID-19 pandemic (2020 and after). A cross-sectional survey was employed most (6) as the research method for conducting the study. The number of States where the studies were conducted is four (4) States (out of the thirty-six States in Nigeria) and the Federal Capital Territory. Three out of the ten included studies were conducted in Lagos State, followed by two in Osun State. The focus participants were mostly community pharmacists as 70 % of the included studies either targeted community pharmacists or community pharmacists and other participants.

### 3.1 Theme 1: adoption and acceptance of telepharmacy growing

There are identified available technological applications for telepharmacy services nationwide, like NIGCOMHEALTH, Mobidoc, and Kangpe (12). The promising potential of adopting telemonitoring services among chronic disease patients was observed in Enugu State (13). The FCT has about half (48%) of out of 381 sampled community pharmacy clients being aware of telepharmacy services (14).

Most community pharmacists, 82 (96.5%) of the respondents, were aware of the importance of telepharmacy and 54 (63%) are practicing it (15). The unaware showed a willingness to engage in telepharmacy services (15). Six sampled cardiologists for interview and 9 sampled community pharmacists for focus group discussion show positive attitudes towards remote monitoring of patients in Lagos State (16). However, a slightly negative attitude of 82 community pharmacists was reported in Osun State, with mean weighted average of 2.4 out of 4.0 after 0–2 scores were taken to connote negativity (17).

*“This is the future, clinics are too busy, the app is good”*—IDI cardiologist (4)

*“Can you compare it? Walking to the pharmacy whenever it is convenient or spending the whole day at LUTH (a teaching hospital)”*—FGD pharmacists (4)

*“Pharmacists are accessible. For doctors, you have to go through procedures before you can see them. Most of the patients do not go to the hospital, they do not get a prescription. We are the ones trying to see what we can do to help them.”*—IDI pharmacist (16)

### 3.2 Theme 2: telepharmacy improving healthcare delivery and patient outcomes

A telepharmacy services project entailing task-shifting of care by cardiologists to community pharmacists led to retention of care of about three-quarters of hypertensive patients out of the 30 interviewed (4, 16). Satisfaction with care and medication adherence were reported. The cardiologists, pharmacists, and patients were all satisfied with the care. Seventy percent of sampled 403 community pharmacy clients in Anambra State reported acceptability of the care being rendered using telepharmacy also (18). In Osun State, 61 diabetes patients’ in the intervention group of a study had improvement in their glycemic control and medication adherence by the use of Short Message Services (SMS) reminders (19).

### 3.3 Theme 3: there exist workforce capacity and training challenges in telepharmacy use

In Lagos State, staff shortages that are detrimental to telepharmacy implementation were reported in the 5 private community pharmacies which engaged in a telepharmacy project (16). In Anambra State, 118 sampled community pharmacists have moderate knowledge (79%) of telepharmacy (18). Lack of monetary motivation, lack of software, and operational difficulties were considered implementation barriers (18). There was another report of mHealth tools being suitable for improving healthcare workers’ efficiency but there is a need for training them on their use (12).

### 3.4 Theme 4: inadequate technology and infrastructure as a barrier for telepharmacy use

In Lagos State, the mHealth app in a telepharmacy project was reported not to be user-friendly (4). In FCT, four out of every five community pharmacy clients of the 381 sampled respondents purchase data for internet connection out of pocket while six out of every hundred do not have access to internet (14). Unreliable internet connectivity was reiterated in other studies (4, 15, 16). A nationwide unreliable and poor internet connectivity—more pronounced in rural areas—was reported, also (12). Data privacy concerns and power supply instability were reported (12). Poor patronage, high installation costs, and lack of ICT resources were other identified challenges facing telepharmacy (15). Telephone interviews were reported to be possibly more used than videoconferencing because of the challenges (15).

*“We have to look for a way to do it without necessarily having internet, because we live in a country where the best internet provider cannot guarantee you internet service 24/7”* (IDI cardiologist) (4).

### 3.5 Theme 5: financial constraints are affecting access and sustainability of telepharmacy use

In Lagos State, it was reported that patients faced financial constraints that hindered payment for telepharmacy services and/or medications (4, 15, 16). Economic recession was reported as a factor responsible for this. There was a report of direct and indirect healthcare cost burden and limited resources (12). Recommendations of community pharmacists to patients to visit hospitals were noticed to be rejected by some, with financial constraints being one of the reasons cited. In Osun State, business size and performance were reported to be hindering telepharmacy implementation (17). Healthcare financing policy reform was suggested (17). In Enugu State, two out of every five of the 335 sampled community pharmacy clients showed willingness to pay for telemonitoring services (13).

*“Some paid. Some did not. I thought I would rather add some money than start dragging them [patients] for money. So, when OMRON was asking me for data, I was like let us leave data out of this”*—IDI pharmacist (4).

### 3.6 Theme 6: there exist policy and regulatory gaps for telepharmacy use

A nationwide narrative review reveals that regulatory frameworks have not been adapted to support telemedicine (telepharmacy inclusive) and telepharmacy services cannot be governed effectively (12). However, it was stated that measures are on the way to address the challenge and the Federal Ministry of Health is one of the agencies facilitating the effort to develop policy document (12).

### 3.7 Theme 7: telepharmacy has the potential to expand healthcare services in rural area

There was a nationwide report of almost half of rural dwellers in need of access to healthcare resources, medicine inclusive (12, 20). It was noted that a large percentage of Nigerians are rural dwellers and quality care is either lacking or almost non-existent (20). There is also shortage of healthcare workers which has resulted in task-shifting of duties of specialists to Community Health Extension Workers (CHEWs) in rural areas (12). CHEW are less-trained healthcare workers lacking highly specialized skills for patients’ care. Therefore, telepharmacy can help ensure rural dwellers have access to specialists while ensuring no need of travelling to urban healthcare facilities where specialists can be found (12).

## 4 Discussion

In Nigeria, COVID-19 has led to a new level of adoption of telepharmacy innovation and studies on telepharmacy were reported during or after the COVID-19 pandemic. Nevertheless, studies on telepharmacy are lacking in many States, implying telepharmacy is still probably at an infant age in Nigeria. Lagos State is leading the way in studies of telepharmacy services, likely because it is Nigeria’s economic capital and has the highest internally generated revenue among all the States (21). It is home to many international and local organizations (22, 23) and higher institutions of learning (24). Most of the studies being cross-sectional may point to the need for obtaining baseline data for implementation of the emerging telepharmacy concept in Nigeria. The business-oriented nature of community pharmacists might have resulted in telepharmacy studies focusing on them (25), and not on hospital pharmacists. None of the studies include hospital pharmacists, most likely because of the shortage of manpower to carry out such duties as telepharmacy services (26). It is expedient that hospital pharmacies are adequately manned and hospital pharmacists’ clinical skills tapped for expanded access to pharmacy services to rural dwellers, especially patients from rural areas attending the hospital.

### 4.1 Analysis of telepharmacy in Nigeria across WHO building blocks

#### 4.1.1 Health service delivery

Technological applications are seldom utilized for rendering telepharmacy services. Telephone services, like SMS, look easier to use than the technological app (19). There is a limit to which SMS can be used in telepharmacy services. Those that have video, are preferable in telepharmacy services to ensure patients are seen, communicated with, and counselled appropriately (27). Therefore, efforts should be geared towards large scale implementation of technological apps.

The high rate of retention of care and improvement in medication adherence (19) experienced with telepharmacy services can be due to reported patient satisfaction with care received from community pharmacists while using telepharmacy services (4). The convenience that can be experienced in telepharmacy services is also a reason for satisfaction with the care rendered (4, 16). Such conveniences include having services delivered at home or nearby, avoiding long waiting times in hospitals, and reducing the expenses incurred for receiving services, among others. Therefore, telepharmacy services may be more appreciated by people living in rural areas and chronic disease patients (1, 28, 29).

#### 4.1.2 Health workforce

There is a shortage of healthcare workers in Nigeria (30, 31), which makes telepharmacy services a necessity for providing quality pharmaceutical services. This is especially necessary because of the task-shifting being experienced in healthcare, especially in rural areas where there is a lack of highly qualified healthcare professionals like pharmacists (32, 33). Therefore, there is a need to implement telepharmacy and train the available pharmacists on telepharmacy services. Furthermore, healthcare facilities in rural areas can be connected to healthcare facilities in urban areas for telepharmacy services, thereby ensuring patients in the rural healthcare facilities can have access to the expertise of pharmacists in urban healthcare facilities.

The fact that some pharmacists are aware of the importance of telepharmacy services and show a positive attitude toward it will help in its successful implementation (15, 18). For those who showed a negative attitude toward telepharmacy services (17), the factors responsible for their negative attitude, like lack of software, moderate knowledge, and operational difficulties, should be addressed.

#### 4.1.3 Health information system

The main challenges that may hinder effective telepharmacy services are unreliable internet connectivity and unstable power supply (4, 12). These may require governmental intervention. For telepharmacy services to be effective, a stable and reliable network and internet connectivity are essential (1, 28, 34). Technological applications that support low bandwidth can be designed and used in places with unstable internet. Meanwhile, telephone interviews, which have been observed to be in use (19), can be a good starting point for telepharmacy services across the nation before the full digitalization of telepharmacy services.

For full digitalization of telepharmacy services, it is important to ensure efficient interoperability of technological applications (35). The applications must be designed in an easy-to-use manner. Patients and healthcare workers in limited-resource settings, like rural areas, must be factored into technological applications design based on the available resources in the area and the level of literacy. Technological applications that can connect urban and rural healthcare facilities for telepharmacy services to patients will be important for increased health care access.

#### 4.1.4 Access to medicines

The difficulty with access to medicines and healthcare resources is mostly experienced by rural dwellers (36). Therefore, it is important that the primary health care of the country be strengthened. Pharmacists in urban areas can engage in telepharmacy services, which will create opportunities for sourcing and delivery of drugs to rural dwellers. Pocket-friendly medicines, which are cost-effective, should be a source for this category of people for affordability reasons.

In addition, access to safe and effective medicines can be improved using telepharmacy (29). Registered and regulated pharmacy outlets that make use of telepharmacy can be established. Healthcare facilities and patients in rural areas or places where no such pharmacy is present can order for drugs online from the established pharmacy outlets for delivery to them. This will ensure that access to safe and effective medicines at a low cost will be sustained.

#### 4.1.5 Health systems financing

Telepharmacy can help ensure sustained healthcare financing through cost control and payment methods (37, 38). In the use of telepharmacy, the number of pharmacists needed for rendering the services will be reduced, and the services will be able to reach a wider coverage of patients. In addition, payment methods for services can be incorporated into the telepharmacy model at a reduced cost when compared to patients travelling for onsite pharmaceutical services. Therefore, the reduced cost of pharmaceutical services will be sustainable, and the health system better for it.

The nationwide report on financial constraints in accessing quality care (12) shows the need to ensure universal health coverage is achieved in the country. Efforts should be geared towards ensuring the populace has health insurance, irrespective of their locations. This will ensure the efforts towards telepharmacy services are not a waste, as the populace will be able to have access to care and medicines. Community pharmacies, being the first point of call for healthcare for people, can be incorporated into the health insurance scheme as stakeholders and considered as primary healthcare facilities.

#### 4.1.6 Leadership and governance

Recently, efforts of the Federal Ministry of Health towards regulating telehealth in Nigeria have resulted in the development of the Nigeria Digital Health Service Bill, 2025 (39). In addition, the Pharmacy Council of Nigeria, supported by the Federal Ministry of Health, has come up with the Electronic Pharmacy Regulation, 2026 (40). These documents are still awaiting being passed into a nationally recognized legal document that will serve as a regulatory framework and guidelines for integrating digital health technologies into the national healthcare system. The legal backing of these documents will be a good starting point for regulating telepharmacy in Nigeria.

A comprehensive telepharmacy (and telemedicine) policy in Nigeria can be further developed, in the near future, to specifically address telepharmacy. It will be a good starting point for ensuring access to quality pharmaceutical care by the populace, irrespective of location. Such a policy can be enshrined in the National Health Policy. After its development, it is necessary to ensure that there is adequate enforcement of the policy.

### 4.2 Comparison with other lower-middle income countries (LMICs)

There have been efforts in other LMICs in Sub-Saharan African countries towards integrating telepharmacy into their healthcare systems and proffering solutions to challenges faced in its implementation (41). However, there have been no large-scale implementation in these countries. Unlike Nigeria, which is primarily focusing on community pharmacy for telepharmacy implementation, Ghana has used the hospital pharmacy-based and disease-specific approach (42). In addition, a bottom-up approach was recommended for telepharmacy implementation in Ghana (43). Telepharmacy has been used for supporting contraceptive decision-making in Kenya (44). Uganda has an appreciable level of telepharmacy practice and infrastructure, but lack human resources (45). South Africa adopted telepharmacy within established telehealth systems during COVID-19 (46). These countries, like Nigeria, are facing similar challenges in scaling up telepharmacy services. These include limitation in infrastructure like financial constraints, regulatory gaps, and workforce gap, among others (44, 46, 47).

Some Asian countries, like China, India and Thailand, have experienced rapid growth in telepharmacy implementation (48), unlike Nigeria and countries in Sub-Saharan Africa. In China, telepharmacy model has been implemented, with examples being e-pharmacy platforms (49) and remote pharmacy services for primary care (50). E-pharmacy platforms make provision for uploading of prescriptions, consultation with a pharmacist, and home delivery of drugs (49). The remote pharmacy services for the primary care telepharmacy model connect primary healthcare facilities with hospital pharmacists for the validation of prescriptions and appropriate guidance (50). The telepharmacy landscape in a few Asian countries, like China and Japan (51–53), has regulatory backing, while the regulatory backing of others, like India and Thailand is still evolving (53–56).

### 4.3 Comparison with international best practice guidelines and lessons learnt

The Nigerian telepharmacy landscape is far from aligning with the World Health Organization (WHO) Global Strategy in Digital Health (57). In addition, it is far from meeting established global telepharmacy framework and best practice guidelines, like the Canadian Telepharmacy Guidelines (58). The Canadian Telepharmacy Guidelines have an implementation framework that centres on people, policy, technology, and quality (58). People involved in telepharmacy are to be trained, pharmacists communicate effectively, and healthcare providers are to take responsibility for the discharge of high-quality pharmacy care and services. Policies for using telepharmacy are to be developed for effective regulation of telepharmacy services and the environment, and ensuring privacy and security. Technology to be used for telepharmacy services is to be in conformity with established regulations, and its design should be appropriate and context specific. Capacity for service provision is to be considered before large scale implementation of telepharmacy services. The use of telepharmacy technology is to be supported by a quality management program with a risk management framework. Therefore, a large-scale implementation of telepharmacy in Nigeria would have to adhere to such best practice guidelines and their framework for effective telepharmacy services.

### 4.4 Readiness for large scale Telepharmacy implementation

As it stands, there may be difficulty in implementing digitalized telepharmacy services in the included States, except in Lagos State which may be partially ready. This is more so because of the various challenges being faced across the six WHO healthcare system building blocks. The challenges are summarized in Table 2. However, a telephone services approach may be a good starting point for the implementation of telepharmacy services, especially in other States other than Lagos State. Studies on telepharmacy can be conducted in other States where such studies have not been conducted before. A telepharmacy (and telemedicine) policy is needed to speed Nigeria’s readiness for telepharmacy through regulation and governance of the services across the nation.

### 4.5 Strategies for enhancing telepharmacy in Nigeria

Having analyzed the situation of telepharmacy use in some States in Nigeria, suggestions of some strategies for enhancing telepharmacy in Nigeria are highlighted in Table 3. The suggestions are targeted towards either the government or professional organizations, as applicable.

**Table 3:** Suggested strategies for enhancing telepharmacy in Nigeria.

WHO building block	Challenges	Strategies	Justifications
Health service delivery	Physicians’ professional takeover concern	Higher institutions should ensure upcoming healthcare professionals live and study together	A culture of teamwork and sameness would have been built in the next generations of healthcare professionals (59)
Health workforce	Healthcare workers shortage and brain drain	Healthcare professionals’ migration policy that stipulate number of years to work in Nigeria before official permission to work in another country	The alarming migration rate of healthcare workers to greener pasture (60, 61) would be reduced
		Government and private healthcare organizations should improve welfarism	It will cut down migration rate and have almost adequate healthcare workers (61)
Health information system	Available digital app not user friendly	Engage digital app developer to build a context-specific app	Context-specific app will be suitable for purpose (62)
	Unreliable internet connectivity	Government should come up with project that will boost internet connectivity across the nation	High internet connection will be made available in rural areas, where telecommunications companies may not focus on due to being profit-oriented (63)
		Telecommunications companies should improve their services	Wider reach of internet connectivity across the nation for effective telepharmacy services (1, 28, 34)
	Unstable power supply	Government should build more electrical (power) grid and ensure adequate maintenance of the existing	Better grids and adequate maintenance of existing grids implies enough power will be generated (64)
		Organisations can make use of solar panel systems as another source of power	A single power supply source would not be relied on for telepharmacy services—alternative source (65, 66)
	Data privacy concern	Educate healthcare professionals on ethical practice of data privacy and confidentiality	Acquiring knowledge and awareness can spur change (67)
	High installation cost	Government and organizations can come up with a loan project for digital implementation with flexible repayment	This will ensure installation cost of digital for telepharmacy does not disrupt other necessary activities and services
	Lack of ICT resources	Government and organizations can come up with a loan project for digital implementation with flexible repayment	This will ensure necessary resources for successful telepharmacy services is obtained
Access to medicines	Difficulty accessing medicine and paying for telepharmacy services due to high medicine cost	Cost-effective medicines procurement	Cost-effective medicine will elicit same therapeutic effect at the barest minimum cost (68)
		Delivery services of medicines to patients by medicine dispatcher	Eliminate the need to travel a distance to access medicine
Health system financing	Financial constraints	Improve the health insurance scheme and ensure coverage of all	Financial security will be obtained (69, 70)
Leadership and Governance	No adaptable regulatory framework	A government policy on telepharmacy (and telemedicine) should be promulgated and enforced	It will showcase the seriousness of the government in elevating telepharmacy (and telemedicine) for inclusive healthcare for all.

### 4.6 Limitation

This is a narrative review and has the limitation of not being inclusive of all available literature. The use of two keywords may have excluded some relevant studies that adjacent terminology might have retrieved. In addition, quality appraisal of included studies was not conducted. Therefore, a well-structured review, like a systematic review, may be needed to proffer a solution to the possibility of missing out on some literature and assessing the quality of included studies.

## 5 Conclusion

Telepharmacy in Nigeria is in its infancy and emerging. A few Nigerian States have reportedly been involved in it. Community pharmacists are participating it. Lagos State, in particular, the economic capital of the country, is leading the course and shows comparatively greater preparedness for large-scale implementation of telepharmacy. Despite the reported benefits, the analysis of telepharmacy using the World Health Organization’s six health system building blocks also shows varying challenges. These challenges make some States in the country unready for large-scale telepharmacy implementation. The challenges can be addressed systemically through governmental, professional, and institutional contributions towards solving them. After solutions have been proffered, then Nigeria will be fully equipped to offer telepharmacy services and ensure quality healthcare for all in all places.
