# Supplementary material for: Telepharmacy in Nigeria: a narrative review
Source: Front Public Health. 2026 Jun 17;14:1843197. doi: 10.3389/fpubh.2026.1843197 (PMC13318991; doi:10.3389/fpubh.2026.1843197)
Supplement: Supplementary file 1 [file Table_1.DOCX]

**SUPPLEMENTARY FILE**

**Appendix 1: Search Strategy**

**(last searching on May 8, 2025)**

| **Search Code** | **Terms** | **Results** | | | | |  |
| --- | --- | --- | --- | --- | --- | --- | --- |
|  |  | **CINAHL Plus** | **EMBASE via Ovid** | **PsychINFO via Ovid** | **Scopus** | **Web of Science** | **Google Scholar** |
| **Population (P)** | | | | | | |  |
| #1 | Nigeria | 12,928 | 63,042 | 7,520 | 132,749 | 194,899 |  |
| **Intervention (I)** | | |  |  |  |  |  |
| #2 | Telepharmacy | 125 | 560 | 22 | 483 | 349 |  |
| **Combined P and I** | | | | | | |  |
| **#3** | **#1 AND #2** | **0** | **2** | **1** | **1** | **3** | **28** |

Search string: “Nigeria” AND “Telepharmacy”

Keywords were broadly searched with no application of filters.
